# Reliability and safety of miniscrew insertion planning with the use of lateral cephalograms assessed on corresponding cone-beam computer tomography images

**DOI:** 10.1093/ejo/cjae003

**Published:** 2024-02-16

**Authors:** Giuseppe Perinetti, Jasmina Primozic

**Affiliations:** Private Practice, Nocciano (PE), Italy; Department of Dental and Jaw Orthopaedics, Faculty of Medicine, University of Ljubljana, Ljubljana, Slovenia

**Keywords:** miniscrews insertion planning, lateral cephalogram, miniscrew insertion safety

## Abstract

**Background/Objectives:**

Recently, lateral cephalograms have been proposed for guided miniscrew insertion planning. Therefore, the aim was to assess the reliability and safety of such planning on corresponding cone-beam computer tomography (CBCT) images.

**Materials/Methods:**

Intraoral scans, lateral cephalograms, and CBCT images of 52 subjects (even sexes distribution), aged 15.1 ± 2.5 years, were included. Miniscrew (*n* = 104) insertion planning was performed using lateral cephalograms superimposed on the maxillary intraoral scans, while the assessment of their bicortical placement, length in bone, contact with adjacent teeth, incisive canal, and nasal floor perforation was done on corresponding superimposed CBCT images. Moreover, maxillary incisor inclination, crowding, and the maxillary intercanine width were measured.

**Results:**

The overall miniscrew length in bone was 7.2 ± 1.3mm. Bicortical placement was seen in 58.7% of the sample (38.5% of subjects). Incisive canal and nasal floor perforation was seen in 25% and 21.2% of subjects, respectively. No contact of the miniscrew with adjacent teeth was recorded. A negative significant interaction was seen between the miniscrew length in bone, the percentage of total miniscrew length and maxillary anterior teeth crowding (β, −0.10, *P* = .047 and β, −0.90, *P* = .006, respectively). Moreover, a positive significant interaction was seen between the incisive canal perforation and maxillary anterior teeth crowding (OR = 1.32, *P* = .021).

**Limitations:**

Exclusion of subjects with impacted teeth.

**Conclusions:**

Miniscrew insertion planning using lateral cephalograms, despite being safe in preventing contact with adjacent teeth, is limited in achieving bicortical placement and insufficient in completely avoiding incisive canal and nasal floor perforation.

## Introduction

The increasing use of miniscrews as anchoring devices in various orthodontic [1–3] or even orthopaedic [4–6] treatments has evidenced, aside from the advantages, several pitfalls related to their stability and possible deleterious side effects to the surrounding structures [7–13]. Among other insertion sites, the hard palate, especially its anterior and paramedian region, has been advocated as the safest region for miniscrew insertion [7, 8, 10, 11, 13–15].

Despite direct ‘free-hand’ insertion following general anatomic guidelines [7, 16–20] is possible, such a procedure does not take into account the individual variability of palatal morphology [7, 8, 14, 21–23]. Therefore, pre-surgical miniscrew insertion planning has been advocated using two-dimensional [24, 25] or three-dimensional [26, 27] radiographic recordings (i.e. lateral cephalograms and cone-beam computer tomographs—CBCTs, respectively) combined with maxillary digital models.

Pre-surgical insertion planning may contribute to the stability of the miniscrews by increasing the length of the miniscrew in bone with accurate bicortical placement [9, 24, 25], and to a more precise miniscrew placement, avoiding incisive canal [26], nasal floor perforations, or collision with adjacent structures [27]. Moreover, such an approach may implement a one-visit protocol according to which appliances are mounted soon after miniscrew insertion.

Because of the lower radiation risk, previous investigations [27–29] recommended lateral cephalograms for miniscrew insertion planning, irrespective of the limitation related to the two-dimensional nature of the image. However, due to the overlapping of structures on the lateral cephalograms, challenges related to surrounding tissue damage should be carefully addressed. Despite the clinical relevance, there is a paucity of data concerning the reliability of miniscrew insertion planning by the use of lateral cephalograms, mainly related to parameters affecting miniscrew stability (bicortical placement and length of the miniscrew in bone) and safety of their insertion (incisive canal and nasal floor perforation). Similarly, no data regarding potential patient and occlusal parameters that may affect the reliability of miniscrew insertion planning using a lateral cephalogram has been reported to date. Therefore, the study aimed to assess the reliability and safety of miniscrew insertion planning using lateral cephalograms as assessed on corresponding CBCT recordings.

## Material and methods

Ethical approval for the study was gained by the Slovenian Ethical Research Committee (Ref. 0120-526/2022/4), along with the informed consent of the included subjects to use their anonymized data.

Consecutive healthy patients with permanent dentition were referred to the University Medical Centre of Ljubljana (Slovenia), of whom pre-treatment three-dimensional intraoral digital scans, digital lateral cephalograms, and at least maxillary CBCT images were available as part of their diagnostic records, were screened. Subjects with cysts or tumours of the maxillary region, cleft lip, and palate, as well as craniofacial syndromes, impacted teeth or missing incisors, and facial asymmetry were excluded. Lateral cephalograms were acquired at 300 dpi with a reference ruler, and images with low quality, blurred, unclear details or with evident double contours at the mandibular or occlusal planes or ear rods were discarded (minimal double contours were accepted).

The final sample consisted of 52 subjects aged 15.1 ± 2.5 years (26 females and 26 males, age range 12–22 years). The intraoral scans of the subjects’ dental arches were obtained using the TRIOS 3 intraoral scanner (3Shape, Denmark), and the lateral cephalograms and CBCT images (with an isotropic voxel size of 0.3 mm) were acquired using KaVo OP 3D Pro imaging system (KaVo Dental GmbH, Germany).

### Assessment of dental parameters

Among the dental parameters, maxillary incisor inclination, maxillary anterior teeth crowding, and maxillary intercanine width were measured. The incisor inclination was measured on lateral cephalograms as the angle between the palatal plane (through the spina nasalis anterior and posterior) and the tooth vertical axis (through the incisal edge and apex). Crowding was assessed on digital scans by measuring the mesiodistal widths of the maxillary canines and incisors and the dental arch length of the left and right anterior segment (from the mesial approximal surface of the first premolar to the midline, bilaterally). Maxillary anterior teeth crowding was then calculated as the difference between the sum of the mesiodistal widths of anterior teeth and the sum of the bilaterally available space in the dental arch. The maxillary intercanine width was measured as the linear distance between the contralateral cusps of the maxillary canines. All the analyses of the dental parameters were performed using Dolphin Imaging & Management Solutions (Patterson Dental Holdings, Inc., USA).

### Miniscrew insertion planning

The lateral cephalogram was properly rescaled according to the reference ruler and aligned on the mid-sagittal plane of the corresponding dentition scan. Careful superimposition (manual rotation and movement) of the cephalogram was performed until the best possible alignment with the contours of the teeth (maxillary and mandibular) and soft palate was obtained. Several reference points have been considered, including the maxillary incisor edge, first or second molar cusps, and the contour of the palatal vault. The two recordings were locked in the registered position for miniscrew insertion planning.

Virtual miniscrews of 7, 9, 11, and 13 mm in length and 2 mm in diameter resembling commercial products (codes 003-2007, 003-2009, 003-2011, and 003-2013, Leone, Sesto Fiorentino, Italy), were used in the study. The insertion planning protocol of two miniscrews complied the following rules: (i) the miniscrews were inserted in the anterior palatal region at the third palatal rugae level, (ii) paramedian (bilaterally 4 mm from the mid-sagittal plane whenever anatomically possible), (iii) parallel to each other, at an adequate (more than 0.5 mm) distance from the projections of teeth roots, and (iv) approximately perpendicular to the palatal vault.

Moreover, an attempt was followed to have the longest miniscrews reaching, but not crossing, the nasal floor line (i.e. bicortical placement). Whenever a double contour of the nasal floor was evident on the lateral cephalogram, the tip of the miniscrew was located between the two contours. The neck of the miniscrew was located through the soft tissue, and when necessary, a minimal part of the lower border of the head of the miniscrew was in contact with the soft tissue (especially in cases of increased height of the palatal vault). This procedure is consistent with a common clinical practice that ensures the abutment of the palatal appliance is properly positioned and secured to the heads of the miniscrews.

After miniscrew insertion planning, the lateral cephalogram was made invisible before the superimposition of the corresponding CBCT recording. The lateral cephalogram registration and miniscrew insertion planning were performed by an experienced operator (GP) using Viewbox Software (dHAL Software, Greece).

### Miniscrew position assessment

For each case, the corresponding CBCT image was superimposed and registered with the intraoral maxillary scan (used as a reference in the locked pre-registered position). In detail, a first manual step of approximating the two 3D recordings was performed, followed by an automatic procedure to obtain a best-fit superimposition (closest point algorithm).

The quantitative and qualitative evaluation of 104 miniscrew insertion plans was performed on the aligned CBCT images (used for the three-dimensional miniscrew position assessment). Quantitative assessments consisted of measurements of the amount in bone of the miniscrew as absolute and relative lengths (miniscrew length in bone [mm] and percentage of total miniscrew length in bone) as well as the amount (length) of the miniscrew perforating the nasal cavity (miniscrew perforation length in the nasal cavity [mm], when present).

**Figure 1.**
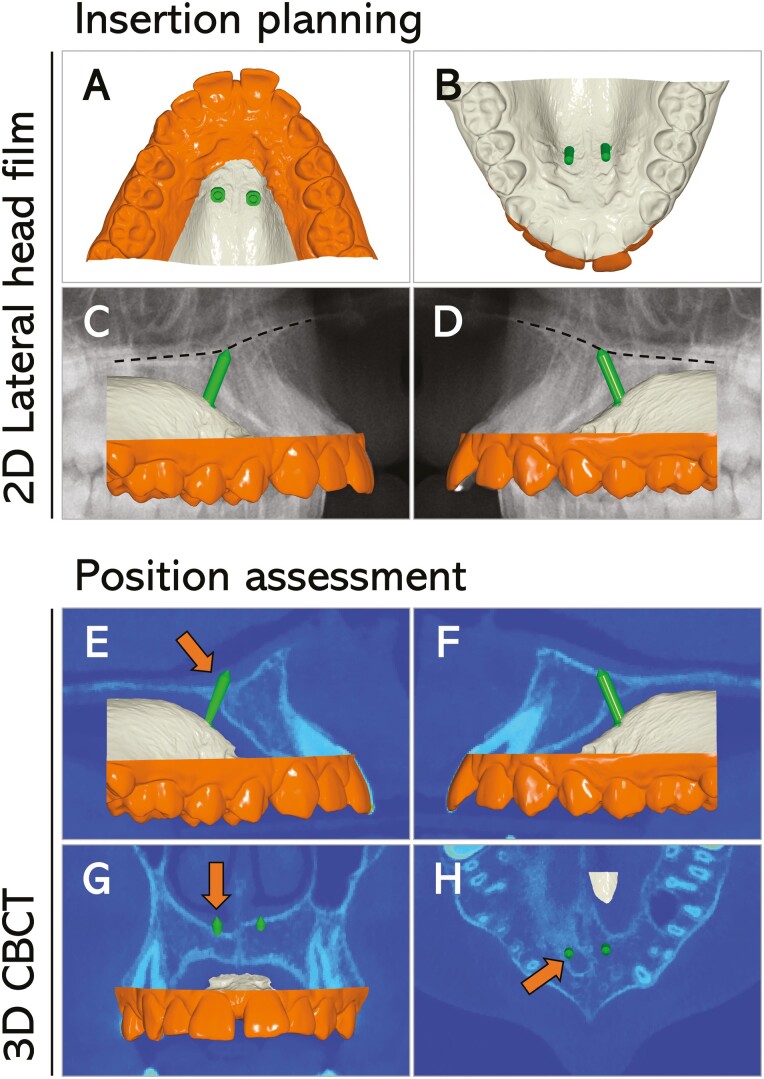
Miniscrew insertion planning using the lateral cephalogram superimposed on the maxillary intraoral scan and the assessment of miniscrews’ positions on the corresponding CBCT image. Miniscrew insertion planning using a subject’s lateral cephalogram superimposed on the maxillary intraoral scan (A–D) and position assessment on the corresponding CBCT image (E–H). Two miniscrews of 9 mm (green) were planned for insertion. Note that the apparent bicortical insertion without any perforations seen on the lateral cephalogram (C, D) was disproved by the assessment on the CBCT image. In detail, the miniscrew on the right side perforated the nasal floor (E, G; orange arrows) and the incisive canal (H, orange arrow). The asymmetry in palatal bone thickness and incisive canal morphology between the right and left sides (G, H) could not be detected on the lateral cephalogram.

The qualitative evaluation consisted of assessing bicortical miniscrew placement, incisive canal perforation, nasal floor perforation, or miniscrew collision with adjacent teeth roots (count and percentage). When at least the miniscrew tip reached the palatal and nasal cortical bone, bicortical placement was recorded. Incisive canal and nasal floor perforations were recorded when any part of the miniscrew entered the anatomic structure by at least 0.5 mm. The shape of the incisive canal was carefully analysed to avoid any misinterpretation of the nasal cavity. More in detail, sagittal, axial, and frontal views, along with the three-dimensional reconstruction, when needed, were considered in the assessment (Fig. 1).

The CBCT registration and miniscrew position assessment were performed by a second experienced operator (JP), using the Viewbox Software for registration of CBCT recordings with maxillary scans and the assessment of qualitative parameters, while quantitative parameters (measurements) were performed after images were exported into Dolphin Imaging Software.

### Sample size calculation and method error analysis

A sample size of 52 subjects was necessary to detect an effect size coefficient of 0.4 in any comparison with an alpha set at 0.01 and a power of 0.80. Descriptive data were presented as mean values ± standard deviations (SD) for all the continuous data, and counts and frequencies were used for the qualitative data.

Method error for dental parameters measurements (maxillary incisor inclination, maxillary anterior crowding, maxillary intercanine width) and miniscrew length in bone measurements was assessed using the method of moments variance estimator based on the analysis of 15 randomly selected images, repeated 3 months apart, and was calculated as mean and 95% confidence interval. Cohen’s κ was used for testing the repeatability of the qualitative assessments.

### Statistical analysis

The normality of data distribution was assessed using the Shapiro–Wilk test and Q-Q normality plots of the residuals, and the equality of variance among the datasets was tested using the Levene test; parametric methods were used for continuous data. Significance of the differences between the left and right sides in continuous data (miniscrew length in bone, percentage of total miniscrew length in bone, and length of the miniscrew length perforated in the nasal cavity) and quantitate data (bicortical placement, incisive canal perforation and nasal floor perforation) were evaluated by using a paired sample *t*-test and a McNemar’s test, respectively.

Regression analyses were run to assess the factors affecting the amount and accuracy of miniscrew insertion planning using lateral cephalograms, with data for the left and right miniscrews pooled together. Backward Wald multiple linear regression analyses were run, with age, sex, maxillary incisor inclination, degree of maxillary anterior crowding, and maxillary intercanine width entered as independent variables, each with miniscrew length in bone (model 1), percentage of total miniscrew length in bone (model 2), and miniscrew length perforated in the nasal cavity (model 3) as dependent variables. Moreover, backward Wald logistic regression analyses were run with the same independent variables, each with miniscrew bicortical placement (model 1), miniscrew incisive canal perforation (model 2), and miniscrew nasal floor perforation (model 3) as dependent variables.

Statistical analysis was done using SPSS version 27.0 (IBM, USA). A *P*-value of less than .05 was considered statistically significant.

## Results

Method errors were 1.1° (0.8°–1.8°) for maxillary incisor inclination, 0.9 mm (0.6–1.4 mm) for maxillary anterior teeth crowding, and 0.3 (0.1–0.4 mm) for maxillary intercanine width measurements. For the miniscrew lengths in bone measurements, the method errors were 0.2 (0.1–0.4 mm) and 0.2 mm (0.1–0.3 mm) for the right and left insertion sides, respectively.

Overall, miniscrews of 9 mm (*n* = 69, 66.3% of miniscrews) and 11 mm (*n* = 35, 33.7% of miniscrews) were selected for insertion planning. Dental parameters (as mean ± standard deviation) were 109.5° ±9.7° for the maxillary incisor inclination, 2.4 ± 3.9 mm for the maxillary anterior teeth crowding, and 33.5 ± 3.5 mm for the maxillary intercanine width.

The overall miniscrew length in bone ranged from 3.0 mm to 10.8 mm (mean value, 7.2 ± 1.3 mm), the overall percentage of the total miniscrew length in bone ranging from 31.8% to 94.2% (mean value, 74.0 ± 9.4%). Mean values, SDs, minimum and maximum values according to the miniscrew insertion site (right or left), and the difference between sides are summarized in Table 1.

**Table 1.** Miniscrew length in bone, percentage of total miniscrew length in bone, and length of the miniscrew perforated in the nasal cavity according to the insertion side.

Parameter	Mean ± SD		Min-Max		*P*-value
	Right	Left	Right	Left	
Miniscrew length in bone (mm)	7.1 ± 1.3	7.2 ± 1.4	3.5–9.8	3.0–10.8	.713; NS
Percentage of total miniscrew length in bone (%)	73.6 ± 9.8	74.3 ± 11.3	31.8–89.1	33.3–94.2	.609; NS
Miniscrew perforation length in the nasal cavity (mm)	0.2 ± 0.6	0.1 ± 0.4	0–1.9	0–2.1	.256; NS

*P*-value refers to the significance of the difference between the right and left sides. SD, standard deviation; Min, minimum; Max, maximum; NS, not statistically significant.

The bicortical placement was seen in 61 miniscrews (58.7% of the sample accounting for 38.5% of subjects). Moreover, 14 miniscrews perforated the incisive canal (13.5% of the sample accounting for 25.0% of subjects) and 12 the nasal floor (11.5% of the sample accounting for 21.2% of subjects). The incisive canal perforation was significantly less frequently (*P* = .048) seen among 9 mm than 11 mm miniscrews insertion planning (8.7% and 22.8% of the sample, respectively). On the contrary, nasal floor perforation was seen significantly more frequently (*P* = .042) among 9 mm than 11 mm miniscrew insertion planning (15.9% and 2.9% of the sample, respectively). The overall miniscrew perforation length in the nasal cavity ranged from 0.5 mm (threshold level) to 2.0 mm (mean perforation value, 1.4 ± 0.4 mm). In none of the cases, collisions with any tooth roots were seen. For none of the above quantitative (Table 1) and qualitative (Table 2) parameters, significant differences were seen between the left and right sides, with the only exception being the bicortical placement, which was significantly more frequent among miniscrews inserted on the right maxillary side (*P* = .027; Table 2).

**Table 2.** Frequency of miniscrew bicortical placement, incisive canal, and nasal floor perforation according to the insertion side.

Parameter	Count (percentage)		*P*-value
	Right	Left	
Bicortical placement	36 (69.2%)	25 (48.1%)	.027; S
Incisive canal perforation	6 (11.5%)	8 (15.4%)	.774; NS
Nasal floor perforation	7 (13.5%)	5 (9.6%)	.754; NS

*P*-value refers to the significance of the difference between the right and left sides. S, statistically significant; NS, not statistically significant.

Results of the multiple regression models for quantitative and qualitative parameters are summarized in Tables 3 and 4, respectively. Regarding quantitative parameters, a negative significant (although small) interaction was seen between the miniscrew length in bone and maxillary anterior teeth crowding (Model 1, β, −0.10, *P* = .047) and between the percentage of total miniscrew length in bone and maxillary anterior teeth crowding (Model 2, β, −0.90, *P* = .006). No significant interactions were seen of the miniscrew perforation length in the nasal cavity. Regarding qualitative parameters, a positive significant interaction was seen between the incisive canal perforation and maxillary anterior teeth crowding (Model 2, OR = 1.32, *P* = .021). More in detail, age and maxillary intercanine width, although kept in the final step, did not reach a statistically significant interaction (*P* = .083 and *P* = .075, respectively). No significant interactions were seen for the bicortical placement and miniscrew nasal floor perforation (Models 1 and 3, respectively).

**Table 3.** Multiple linear backward regression models for quantitative parameters.

Model (dependent variable, adjusted *R*^2^)	β	95% CI	*P*-value
Model 1 (Miniscrew length in bone, 0.058)			
Constant	7.43	6.99–7.88	--
Maxillary anterior teeth crowding	−0.10	−0.19–(−0.01)	.047; S
Model 2 (Percentage of total miniscrew length in bone, 0.124)			
Constant	76.17	73.30–79.04	--
Maxillary anterior teeth crowding	−0.90	−1.53–(−0.27)	**.006; S**
Model 3 (Miniscrew perforation length in the nasal cavity)			
Constant	0.268	--	--

Age, sex, maxillary incisor inclination, maxillary anterior teeth crowding, and maxillary intercanine width were entered as explanatory variables; CI, confidence interval; S, statistically significant.

**Table 4.** Multiple logistic backward regression models for qualitative parameters.

Model (dependent variable, *R*^2^)	OR	95% CI	*P*-value
Model 1 (Miniscrew bicortical placement)			
Constant	0.62	--	--
Model 2 (Miniscrew incisive canal perforation, 0.275)			
Constant	142.7	--	--
Age	0.71	0.48–1.05	.083; NS
Maxillary anterior teeth crowding	1.32	1.04–1.67	.021; S
Maxillary intercanine width	0.82	0.66–1.02	.075; NS
Model 3 (Miniscrew nasal floor perforation)			
Constant	0.268	--	--

Age, sex, maxillary incisor inclination, maxillary anterior teeth crowding, and maxillary intercanine width were entered as explanatory variables; CI, confidence interval; S, statistically significant; NS, not statistically significant.

## Discussion

The present study evidenced a lack of bicortical miniscrew placement upon insertion planning with lateral cephalograms in 41.3% of the inserted miniscrews. Moreover, on average, only approximately three-quarters of the selected total miniscrew length was inserted in bone with such protocol. Regarding deleterious side effects to the surrounding structures, no collision of any miniscrew with adjacent teeth was recorded, while perforation of the incisive canal and nasal floor occurred in 25% and 21.2% of the subjects, respectively. Maxillary anterior teeth crowding was the only parameter among those examined in the present study that was significantly associated with either the length and percentage of total miniscrew length in bone or with miniscrew incisive canal perforation.

Previous studies reported that miniscrews inserted in the palatal area represent stable and safe anchorage support for different kinds of appliances [1, 2, 16, 17, 30, 31]. Recently, Iodice *et al*. [28] reported that no significant differences were seen between the manual and guided (using a lateral cephalogram) miniscrew insertion protocols. This previous study [28] would apparently question the need for radiologic recordings and guided miniscrew insertion planning; however, the authors considered only the differences between the final positions of the miniscrews inserted with either protocol and not factors related to the long-term stability of the miniscrew (i.e. length of miniscrew in bone or bicortical placement) or possible deleterious effects to surrounding tissues.

The present study initially evaluates the issues related to the stability and safety of miniscrew insertion upon planning using lateral cephalograms, according to a posteriori CBCT analysis. From a clinical standpoint, using longer miniscrews was reported as one of the most critical factors in improving their stability upon insertion in the midpalatal region [32]. Moreover, it has been evidenced that miniscrew-assisted rapid palatal expansion with longer miniscrews can increase the amount of maxillary basal bone expansion and improve miniscrew stability [33]. Therefore, the selection of a longer miniscrew insertion, possibly bicortical, appears to be a key factor for treatment success, making reliable digital planning necessary to avoid potential risks of collision/perforation of surrounding structures.

Herein, miniscrews of 9 mm were used in most of the cases, and only approximately one-third of the miniscrews were 11 mm long. In spite of the two different lengths, no significant differences regarding both the frequency of bicortical placement and the percentage of total miniscrew length in bone were seen.

Although bicortical placement was pursued on the lateral cephalogram, up to 41.3 % of the inserted miniscrews failed to be bicortical (Table 2). A lack of bicortical placement might be related mainly to the failure of the two-dimensional lateral cephalogram to detect any asymmetry in the nasal floor contour. Kim *et al*. [19] reported that the palatal bone thickness measured at 1.5–5 mm paramedian at the level of the contact points of maxillary premolars (which was approximately the insertion site used in the present study) appears to match the actual bone thickness depicted on lateral cephalograms of that area reliably. However, the ‘main’ contour seen on the lateral cephalogram might not represent, on an individual basis, the exact location of the nasal floor at the paramedian area where the miniscrew is inserted. Alignment of a two-dimensional lateral cephalogram with a three-dimensional intraoral scan is a procedure with restricted accuracy, although carried out with extreme care. Moreover, by using the lateral cephalogram, the placement of the miniscrew relies on the projection of the miniscrew itself on the cephalogram image.

Maxillary incisor inclination, maxillary anterior teeth crowding, and maxillary intercanine widths were investigated herein to uncover any potential association of these parameters with miniscrew placement based on insertion planning using a lateral cephalogram. These parameters are easily recognizable and measurable by clinicians, and they also correlate with the shape and size of the anterior maxilla.

The present study failed to detect any associations between the examined explanatory dental parameters for bicortical miniscrew insertion. On the contrary, miniscrew length in bone and percentage of total miniscrew length in bone were negatively associated with maxillary anterior teeth crowding. For both parameters, the greater the maxillary anterior teeth crowding, the less miniscrew length (or percentage of total length) is expected in bone. As both bicortical placement [34] and length of miniscrew in bone are key factors for miniscrew stability [33] from a clinical perspective, the use of lateral cephalograms for insertion planning appears not to be accurate enough especially when anterior crowding is present.

Regarding the safety of miniscrew insertion planning with the use of lateral cephalograms, no collisions with adjacent teeth were seen, making planning miniscrew insertion on lateral cephalograms safe and predictable from this point of view and contributing to the avoidance of stability issues related to the miniscrew proximity to roots of the teeth [35].

On the contrary, incisive canal perforation was recorded in about one-quarter of the subjects. Although the incisive canal perforation frequency reached significance comparing insertion of 9 mm and 11 mm miniscrews, with higher frequencies detected among longer miniscrews, the only parameter that significantly increased the odds (by 1.32 times) for incisive canal perforation was maxillary anterior teeth crowding. However, the penetration into the incisive canal was generally limited and mostly seen in the posterosuperior part of the canal. The study by Kim *et al*. [19] reported that at 1.5 mm and 5.0 mm paramedian, at the level of the contact point between maxillary first and second premolars, the incisive canal could be encountered in 60% and 3% of the subjects, respectively The incisive canal was perforated in 13.5% of the sample in the present study, which would be in agreement with previous evidence [19]. Moreover, another recent investigation [36] reported significant variability in the incisive canal morphology, revealing larger canals in older subjects and in subjects with reduced bone thickness. Even though the miniscrew penetration in the incisive canal seen herein has little clinical relevance in terms of patient damage, it might have implications in terms of the stability of the miniscrew. This evidence reinforces the need for a CBCT image for miniscrew insertion planning.

The complications associated with incisive canal perforations either by the use of implants [37] or miniscrews are seldom reported and are associated with miniscrew failure, bleeding, tissue inflammation, and bone necrosis, apparently with complete tissue regeneration upon miniscrew removal [26], no deleterious (sensory loss) long-term effects [37]. Nevertheless, the higher odds of incisive canal perforation associated with anterior teeth crowding, evidenced by the present study, might be related to the shorter anterior palatal area and/or more anterior position of the contact point of maxillary premolars (miniscrew insertion area). Using a CBCT image for miniscrew insertion planning might be beneficial in such cases.

Nasal floor perforations were less frequently seen in the present study (11.5% of the sample) but interestingly more frequently among shorter (9 mm) than longer miniscrews. This apparent controversy might be because shorter miniscrews are usually chosen when planning insertion in thinner palates. Interestingly, in 20% of the cases, nasal floor perforation might be falsely seen on lateral cephalograms, which might have also affected our insertion planning in terms of miniscrew selection length [38]. Furthermore, the individual variations in width and height of the hard palate, as well as its asymmetry or even asymmetries related to the whole head, leading to the apparently greater palatal bone thickness shown on the cephalogram due to blurring [19], might explain the occurrence of nasal floor perforation even when using shorter miniscrews. None of the explanatory parameters tested in the present study reached significance for nasal floor perforations. Nevertheless, perforations in the nasal cavity did not exceed 2 mm of the miniscrew length, which was reported as the threshold for clinically relevant complications [39].

### Limitations of the study

The present study did not include cases with impacted or supernumerary teeth or severe crowding, which probably affected the results related to the frequency of miniscrew collision with tooth roots. Therefore, based on the present study, miniscrew collision with tooth roots upon their insertion (planned by using a lateral cephalogram) could not be excluded in such cases. Moreover, the validity of miniscrew placement using lateral cephalogram images has been assessed virtually on the corresponding CBCT image and not on an additional CBCT recording after *in vivo* miniscrew insertion since, although relevant, such a recording would have been ethically questionable.

### Clinical significance

The use of lateral cephalograms for miniscrew insertion planning has several limitations related to the two-dimensionality of the image. Although collision of adjacent teeth can be confidently avoided by their use even in cases with anterior crowding and a reduced intercanine width, at least in cases with no impacted or supernumerary teeth or severe crowding cases. Therefore, it cannot be excluded that collision with adjacent teeth could be avoided with miniscrew insertion planning using lateral cephalograms in such cases. On the contrary, using a lateral cephalogram for miniscrew insertion planning prevents incisive canal perforation and nasal floor perforation in the majority, but not all, cases. According to the evidence of the present study, it might be hypothesized that the use of a lateral cephalogram for digital planning of miniscrew insertion may have limitations, mainly related to the stability of the inserted miniscrews and patient damage. Finally, the use of lateral cephalograms for miniscrew insertion planning to address miniscrew stability issues, especially in cases (i.e. palatal expansion) when bicortical placement with more considerable miniscrew lengths in bone would be recommended for treatment success, appears to be questionable. This aspect would be of relevance when, on a lateral cephalogram, a double contour is seen at the level of the nasal floor when an anatomical asymmetry may be suspected. Of note, the *in vivo* placement of the miniscrew is almost always subjected to small deviations from the planned insertion despite the use of a guide [40]. Therefore, digital planning using a CBCT image appears clinically relevant as the body and tip of the miniscrew may be planned for placement in a safer position, considering also such unpredictable (even small) discrepancies upon clinical insertion. The use of a CBCT image with a restricted field of view may be a compromise between the reliability of the miniscrew digital insertion planning and radiation exposure.

## Conclusions

Guided miniscrew insertion planning using a lateral cephalogram does not lead to bicortical insertion in approximately 40% of the cases. Only complications related to miniscrew collision with adjacent teeth roots could be avoided, while incisive canal perforations, as well as nasal floor perforations, occur, despite such planning.

## Data Availability

The data underlying this article will be shared on reasonable request to the corresponding author.
